# A Text-Book of Hygiene

**Published:** 1885-08

**Authors:** 


*A Text-Book of Hygiene*, a comprehensive treatise on the principles and practice of preventive medicine, from an American standpoint, by Geo. H. Rohe, M. D., Prof. Hygiene, College of Physicians and surgeons, Baltimore, etc. Baltimore, Thomas & Evans, 1885, octavo—cloth, 324 pages—price $3. We will notice this book at length when we have more carefully examined it.

